# Optimization design of a novel X-type six-high rolling mill based on maximum roll system stiffness

**DOI:** 10.1371/journal.pone.0228593

**Published:** 2020-02-19

**Authors:** Liping Wang, Qingyu Zhu, Hongyang Zhao

**Affiliations:** 1 School of Mechanical Engineering and Automation, University of Science and Technology Liaoning, Anshan, China; 2 School of Mechanical Engineering, Dalian University of Technology, Dalian, China; 3 School of Materials and Metallurgy, University of Science and Technology Liaoning, Anshan, China; University of Genova, ITALY

## Abstract

The present investigation devices a novel X-type six-high (X-6h) mill. In addition, parametric models of different roll layouts such as the four-high (4-h), I-type six-high (I-6h), and X-6h mills are established. Three-dimensional (3D) finite element (FE) contact analysis for a strip rolling process is conducted when the mills are subjected to a constant vertical load of 65 kN. Through comparative analysis of von Mises stress, contact stress and elastic deformation displacement in three roll layouts, the rigidity characteristic of each is obtained, and it is found that the proposed X-6h mill has the largest roll gap stiffness. The influence of different roll diameter ratios on the roll gap stiffness of the roll system is investigated, based on which an optimization design model is built. Further, by taking into account the roll gap stiffness of the roll system as the optimization objective, the optimum diameter ratios of backup roll (BUR) to work roll (WR) of the X-6h rolling mill is achieved via the genetic algorithm (GA) optimization method, obtaining the optimum structural parameters of BUR and WR as well. The reliability of the proposed design is verified by manufacturing a prototype mill which produced magnesium alloy and aluminum alloy strips of high quality.

## 1. Introduction

In the modern steel industry, rolling is widely used in strip engineering applications, which has nearly 90% of market share. There is an urgent need for high-quality strips, which has prompted researchers to investigate the factors affecting thickness and flatness control accuracy of the rolled strip [1–4]. In the strip rolling process, the roll is one of the most important parts for plastically deforming the metal to form a strip, and the quality of strips depends directly on the structure of the roll layouts. When the work roll (WR) is subjected to rolling force, bending deformation of the rolls may occur. The roll gap stiffness of roll systems fluctuates due to the change of the strip rolling direction, especially in the reversible rolling production of the strip. Therefore, the demand for high-stiffness roll layouts is very urgent to obtain high-quality strips. Automatic shape control mainly includes edge drop control, crown control and flatness control in tandem cold rolling mills. In order to reduce the edge drop of the strips and decrease the total strip crown in the industrial rolling process, new-generation high-tech rolling mills are established and have been applied widely for industrial application. Based on the conventional four-high mill and the I-type six-high mill, i.e. six-high universal crown mill (UCM), introduced in Ref.[5,6], a novel X-type six-high (X-6h) mill is proposed.

The bending deformation of the roll system is an important factor that affects the shape of the strip. The deformation involved in the strip rolling process requires a coupling analysis of several models to describe the different deformation phenomenon [7]. During the past decades, many researchers investigated the bending deformation of the roll system, e.g., Hartley P [8], Panton S M [9], and Komori K [10]. At present, methods for studying the bending deformation of the roll system include analytical, influence function, and finite element methods. For example, Sun C G [11] and Dong Q [12] used linear elastic analysis and contact analysis methods to simulate the frame and roll system of a 4-h cold rolling mill respectively, and obtained their deformation, and then calculated the overall rigidity of the rolling mill. Shi X [13] used a 3-D elastic-plastic finite element method to analyze the strip deformation of the cold rolling process in the 4-h mill. The elastic deformation of the roll system and the elastoplastic deformation of the strip were coupled and analyzed according to the contact analysis, obtaining the steel thickness distribution along the width of the strip. A 3D elastic-plastic finite element model of cold strip rolling for the 6-h continuous variable crown (CVC) rolling mill was developed by Linghu K Z [14], and the rolling force distribution was obtained by the internal iteration processes.

There are few studies focused on the influence of roll layouts and roll diameter ratios on the roll gap stiffness. The occurrence of cross buckles does not increase in the UC-4 mill with very small diameter work rolls, but Yasuda did not further compare the influence of different roll diameter ratios on the roll gap stiffness [15]. Aljabri analyzed the total rolling force in detail by changing the roll cross angle and axial shifting roll. However, he did not concentrate on the influence of roll layouts on the roll gap stiffness as well [16].

As for the roll gap stiffness, by knowing the displacement of rolls, the optimum gap between the work rolls can be calculated. Mosayebi [17] proposed a vibration model with two degrees of freedom for a cold sheet rolling mill and calculated the stiffness parameters of different mill elements. Moon [18] put up a modification equation which could eliminate the thickness set-up error as well as the roll-force prediction error, and the measured mill-stiffness values were replaced successively by modified mill-stiffness values until saturated mill-stiffness values were obtained at each stand. After mill-stiffness modification, the exit thickness accuracy was markedly increased, and the operational stability also improved. In [1], intermediate roll shifting (IRS)–induced rigidity characteristics of the six-high universal crown control mill (UCM mill) are established as a key model for the automatic thickness and flatness control system; the vertical and transverse rigidity characteristic curves of the roll stack, elastic deflection of the rolls, and contact stress field between the rolls are extracted. It is found a decrease in IRS values changes the elastic deflection state of the rolls, and this is the formation mechanism of the IRS-induced transverse rigidity characteristic of the UCM mill.

Considering the asymmetric structure of the rolling mill system, Huang [19] established a chatter model by coupling a rolling process model and structure model to describe the asymmetric vibration characteristics, and found that the influence of the asymmetric structure parameters on the stability and critical conditions is noteworthy. In [20], Ma et al. established a structural optimization method for a new Y-type rolling mill, which can solve the multi-objective problem of more complex constraints in engineering.

At present, the mill for rolling strips at home and abroad generally adopts the 4-h or conventional I-6h mill. Through comparative analysis of different mill types with three roll layouts, the roll gap stiffness of the roll system is obtained. Further, the diameter ratios of the WR to the BUR of the X-6h mill are optimized using GA. To verify the high rigidity characteristic of the proposed structure, a prototype mill is manufactured and used for rolling magnesium alloy and aluminum alloy strips.

## 2. Structure description

**Fig 1:**
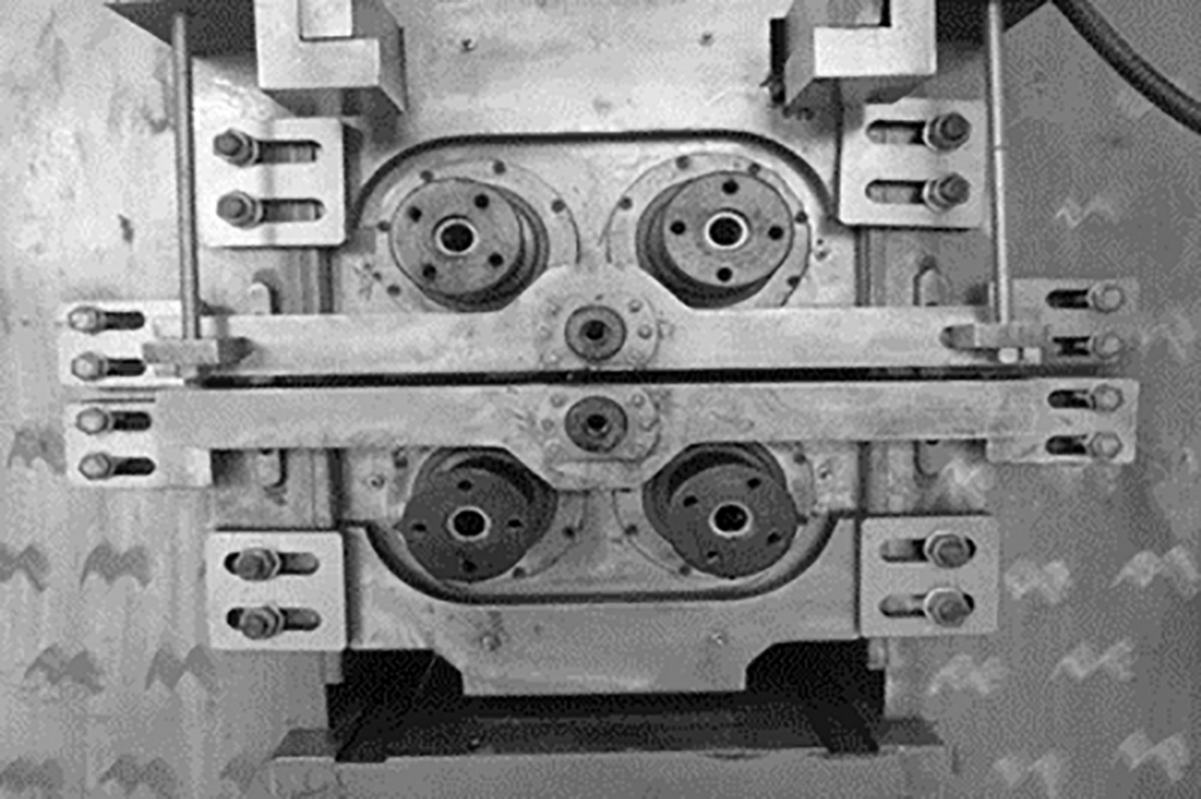
Physical image of proposed X-6h rolling mill prototype.

This section presents the structural analysis of three rolling mills with different roll layouts, and Fig 1 shows a view of the designed X-6h mill.

### 2.1 Comparative analysis of three types of roll layouts

**Fig 2:**
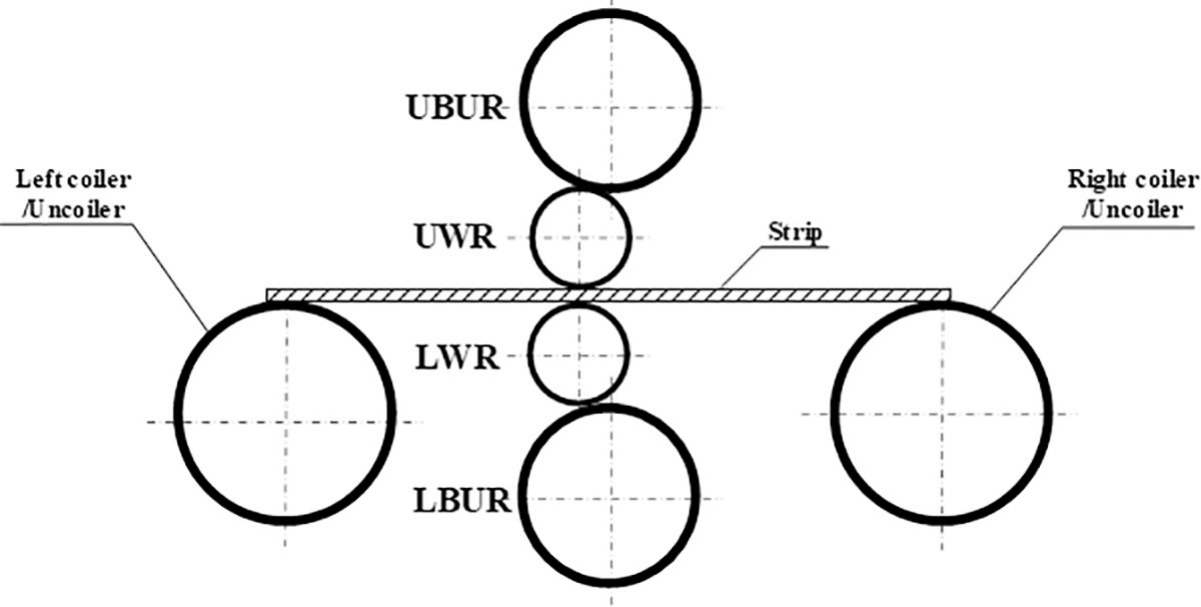
Structure of a 4-h rolling mill.

**Fig 3:**
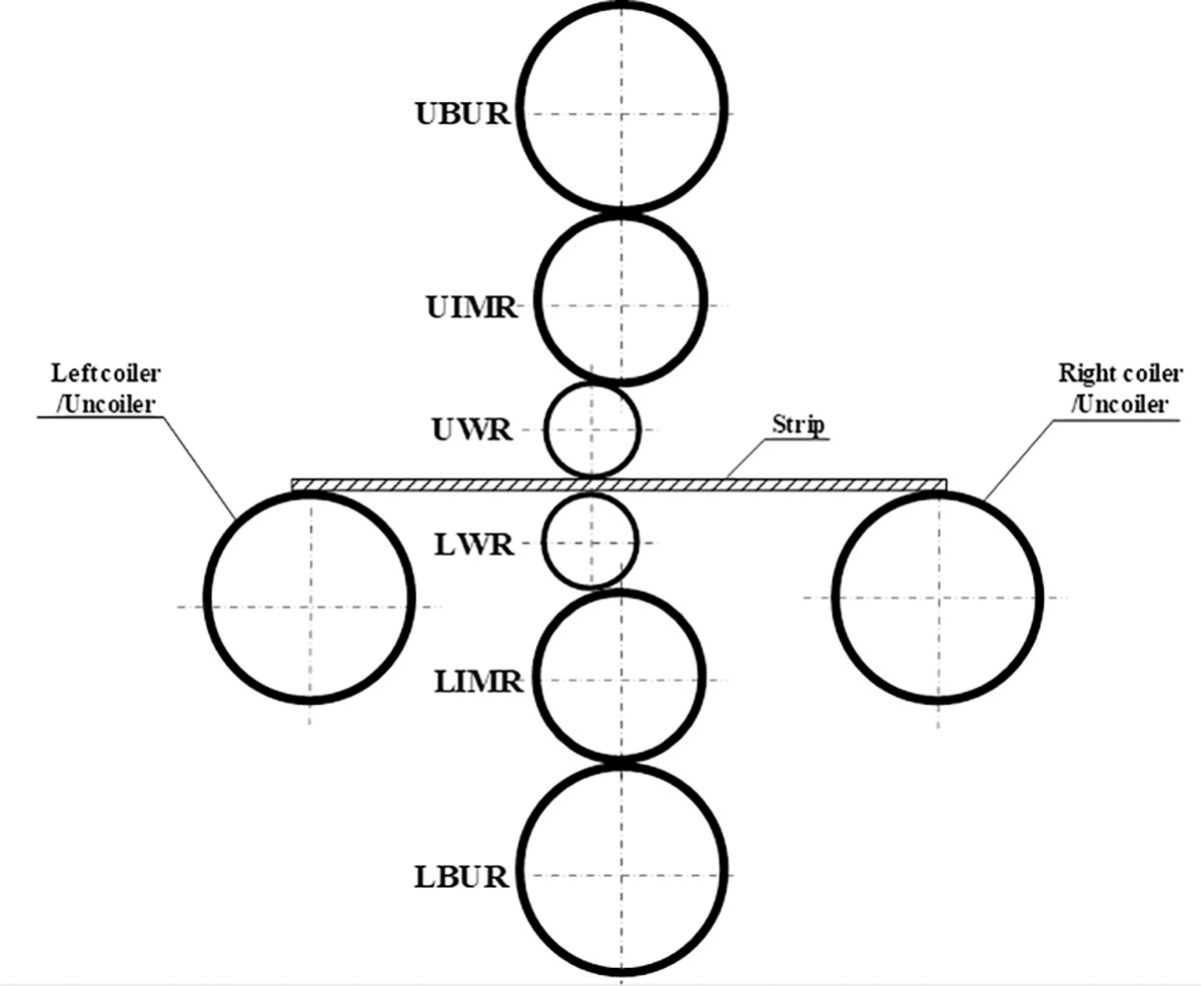
Structure of the traditional I-6h rolling mill.

The roll system structure of the 4-h rolling mill is shown in Fig 2. It consists of the upper backup roll (UBR), upper work roll (UWR), lower work roll (LWR), and lower backup roll (LBR) arranged in a vertical plane from top to bottom. The diameters of UBR and LBR are equal, and those of UWR and LWR are equal; the diameters of BR are larger than those of WR. The roll system structure of the I-6h rolling mill is shown in Fig 3. It is composed of one of each of the following: the upper backup roll (UBUR), upper intermediate roll (UIMR), UWR, LWR, lower intermediate roll (LIMR), and lower backup roll (LBUR) arranged in a vertical plane from top to bottom. The UBUR and LBUR are of equal diameter, as well as the UIMR and LIMR; the UWR and LWR are of equal diameter, and the diameter of BUR is larger than that of IMR, while that of IMR is larger than that of WR.

**Fig 4:**
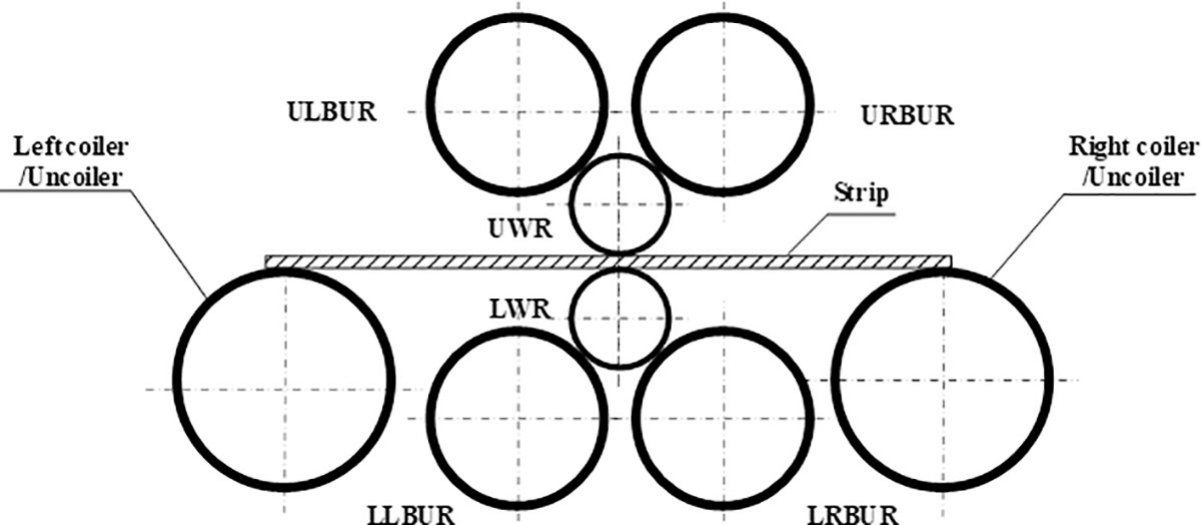
Structure of a novel X-6h rolling mill.

As shown in Fig 4, the roll system of the novel X-6h rolling mill is composed of one upper left BUR (ULBUR), upper right BUR (URBUR), UWR, LWR, lower left BUR (LLBUR), and lower right BUR (LRBUR). These rolls are arranged in a diagonal symmetrical way. The diameters of the ULBUR, URBUR, LLBUR and LRBUR are equal, those of UWR and LWR are equal, and the diameter of BUR is larger than that of WR.

### 2.2 Interaction between rolls

In the roll system of the 4-h rolling mill as shown in Fig 2, the lower surface of UBUR is in contact with the upper surface of UWR. The lower surface of UWR and the upper surface of LWR are respectively in contact with the strip. The lower surface of LWR is in contact with the upper surface of LBUR. There are two contact pairs among the rolls. In the roll system of the I-6h rolling mill as shown in Fig 3, the lower surfaces of UBUR and UIMR are in contact with the upper surfaces of UIMR and UWR, respectively. The lower surface of UWR and the upper surface of LWR are respectively in contact with the strip. The lower surfaces of LWR and LIMR are in contact with the upper surfaces of LIMR and LBUR, respectively. There are four contact pairs between the rolls. In the roll system of the X-6h rolling mill shown in Fig 4, the right lower surface of the LUBUR is in contact with the left upper surface of UWR, and the left lower surface of the RUBUR is in contact with the right upper surface of UWR. The lower surface of UWR and the upper surface of LWR are respectively in contact with the strip. The right upper surface of the LLBUR is in contact with the left lower surface of LWR, and the left upper surface of the RLBUR is in contact with the right lower surface of LWR. There are four contact pairs between the rolls.

### 2.3 Determination of load conditions during rolling process

**Fig 5:**
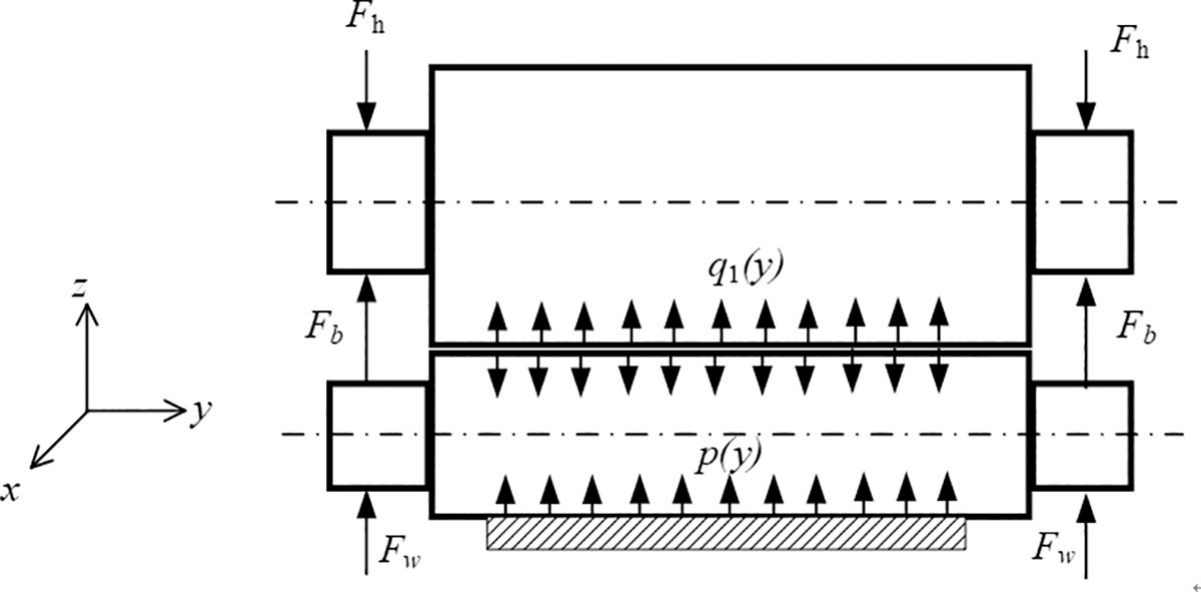
Forces and contact stress in the roll system of the 4-h rolling mill.

**Fig 6:**
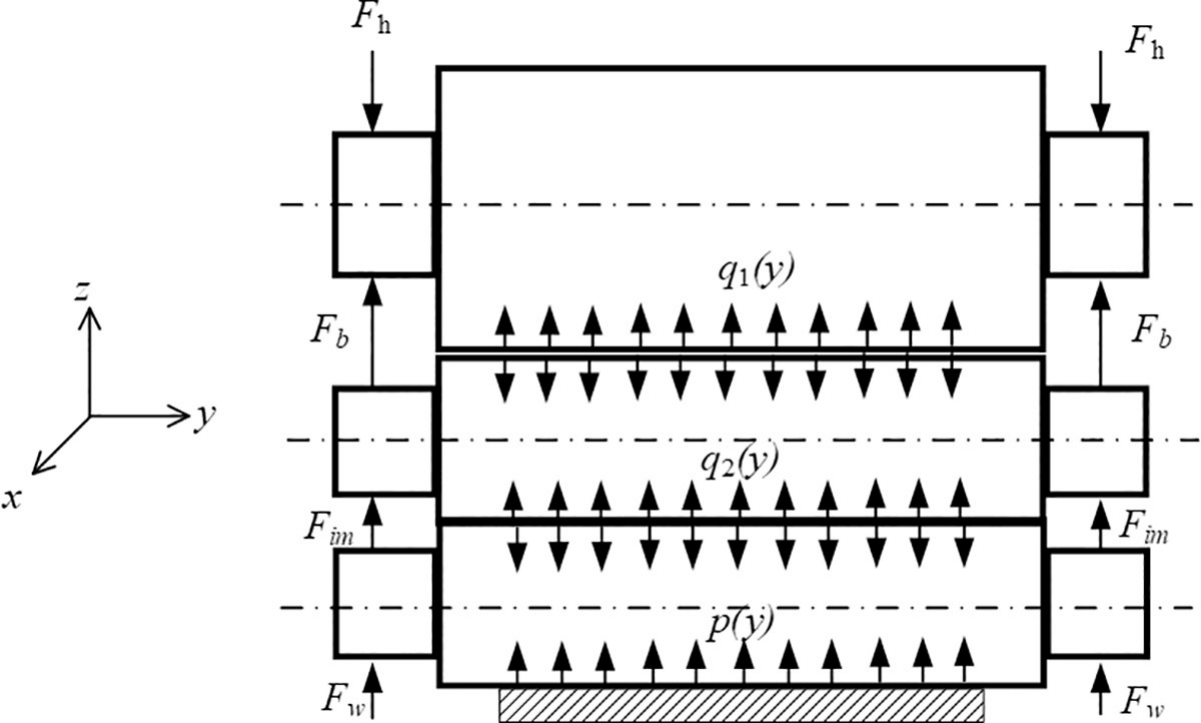
Forces and contact stress in the roll system of the I-6h rolling mill.

**Fig 7:**
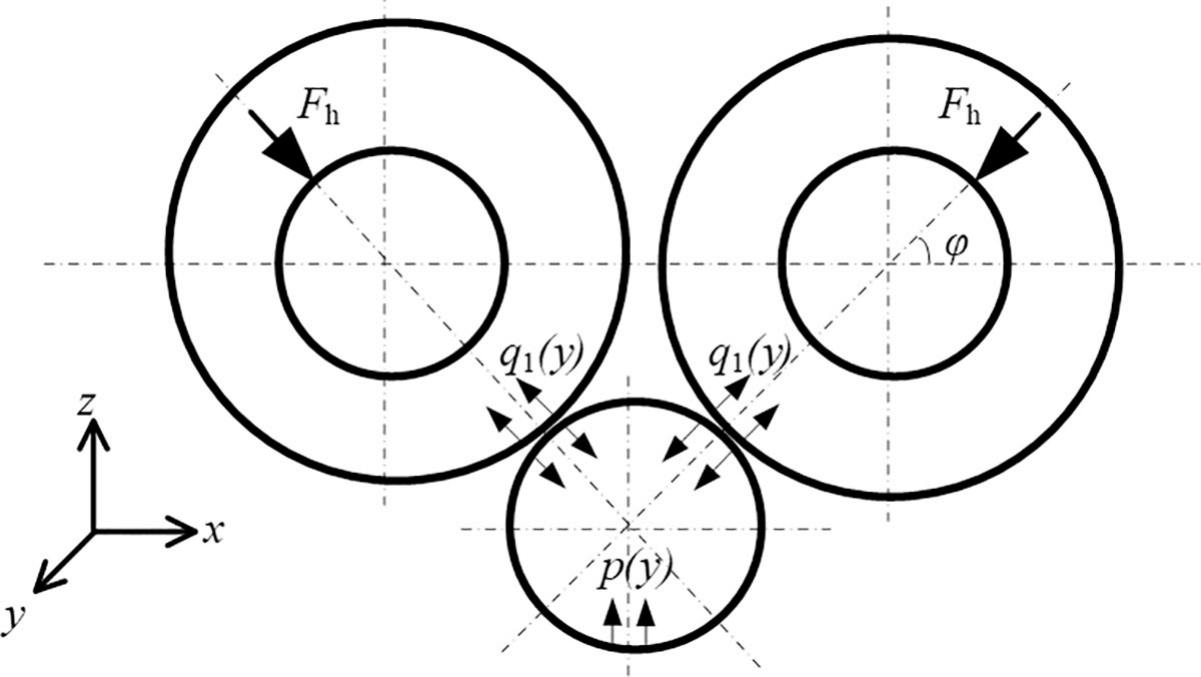
Forces in the roll system of the X-6h rolling mill.

Due to the symmetry of the roll system structure and load conditions, only forces on the upper part of the roll system are analyzed. The forces acting on the roll system of the rolling mill are illustrated in Figs 5–7. In these Figs, *p*(*y*) is the rolling stress per unit width, *q*(*y*) represents the unit contact stress between rolls, *F*_*h*_ is the supporting reaction force, *F*_*w*_ is the bending force of the work roll, *F*_*b*_ is the bending force of the backup roll, and *F*_*im*_ is the bending force of the intermediate roll.

For the 4-h rolling mill, the lower surface of UWR bears rolling force *p*(*y*) during rolling. The rolling force is balanced with support reaction force *F*_*h*_ at the bearing of UBUR, and is expressed by mean unit stress *q*_1_(*y*) applied on the two surfaces, as shown in Fig 5.

For the I-6h rolling mill, the lower surface of UWR bears rolling force *p*(*y*) during rolling. The rolling force is balanced with support reaction force *F*_*h*_ at the bearing on UBUR and expressed by the mean unit stress *q*(*y*). There is contact stress *q*_1_(*y*) between the lower surface of UBUR and the upper surface of UIMR, and there is also contact stress *q*_2_(*y*) between the lower surface of UIMR and the upper surface of UWR, as shown in Fig 6.

For the novel X-6h rolling mill, the lower surface of UWR bears rolling force *p*(*y*) during rolling. The rolling force is balanced with the support reaction *F*_*h*_ of LUBUR and RUBUR, and is expressed by average unit stress *q*(*y*). There is contact stress *q*_1_(*y*) between the right lower surface of LUBUR and UWR, and between the left lower surface of RUBUR and UWR, respectively, as shown in Fig 7.

## 3. Structural design and FEM modeling

In this section, the finite element models of the rolling mills are established according to roll parameters, and the contact stiffness and overall stiffness of the roll systems of the three kinds of rolling mills are calculated. In order to evaluate the structural stiffness of the three kinds of roll layouts, the total rolling force in the present investigation is selected as 65 kN.

### 3.1 Determination of roll parameters

**Fig 8:**
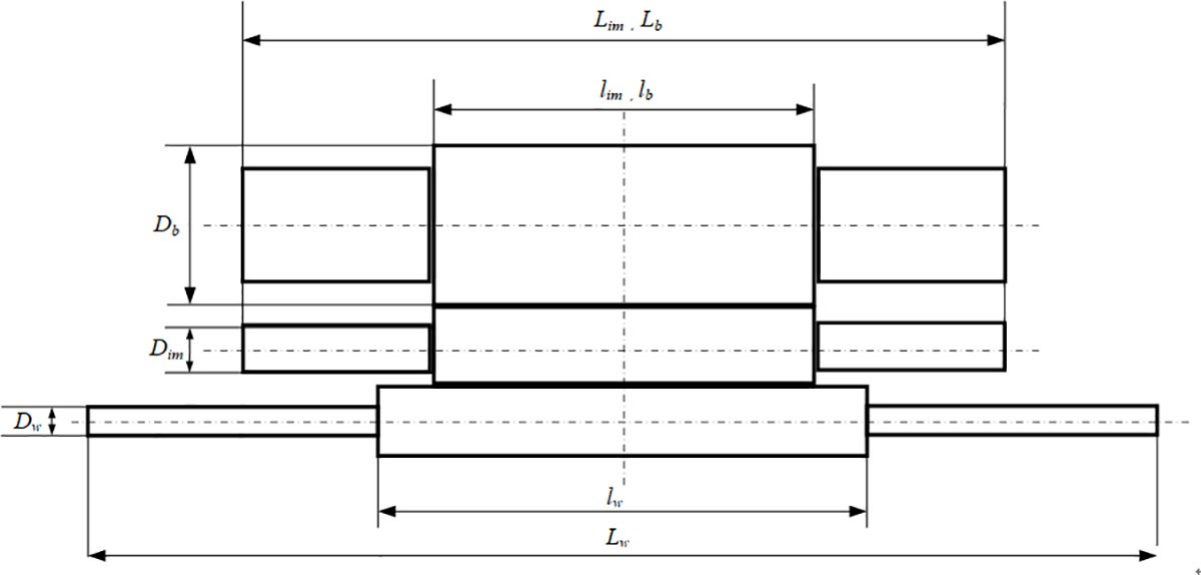
Structural parameters of rolling mills.

The structural parameters of the roll system of the 6-high UCM mill are primarily designed as shown in Fig 8.

In order to compare the stiffness of the three roll layouts, the other two rolling mills, 4-h and X-6h mills, adopt the same sizes of WR and BUR as shown in Table 1.

**Table 1:** Structural parameters of roll system.

Parameters	Value (mm)
Total length of BUR *L*_*b*_	696
Length of roll body of BUR *l*_*b*_	350
Diameter of roll body of BUR *D*_*b*_	150
Total length of IMR *L*_*im*_	696
Length of roll body of IMR *l*_*im*_	350
Diameter of roll body of IMR *D*_*im*_	75
Total length of WR *L*_*w*_	1060
Length of roll body of WR *l*_*w*_	450
Diameter of roll body of WR *D*_*w*_	60

**Fig 9:**
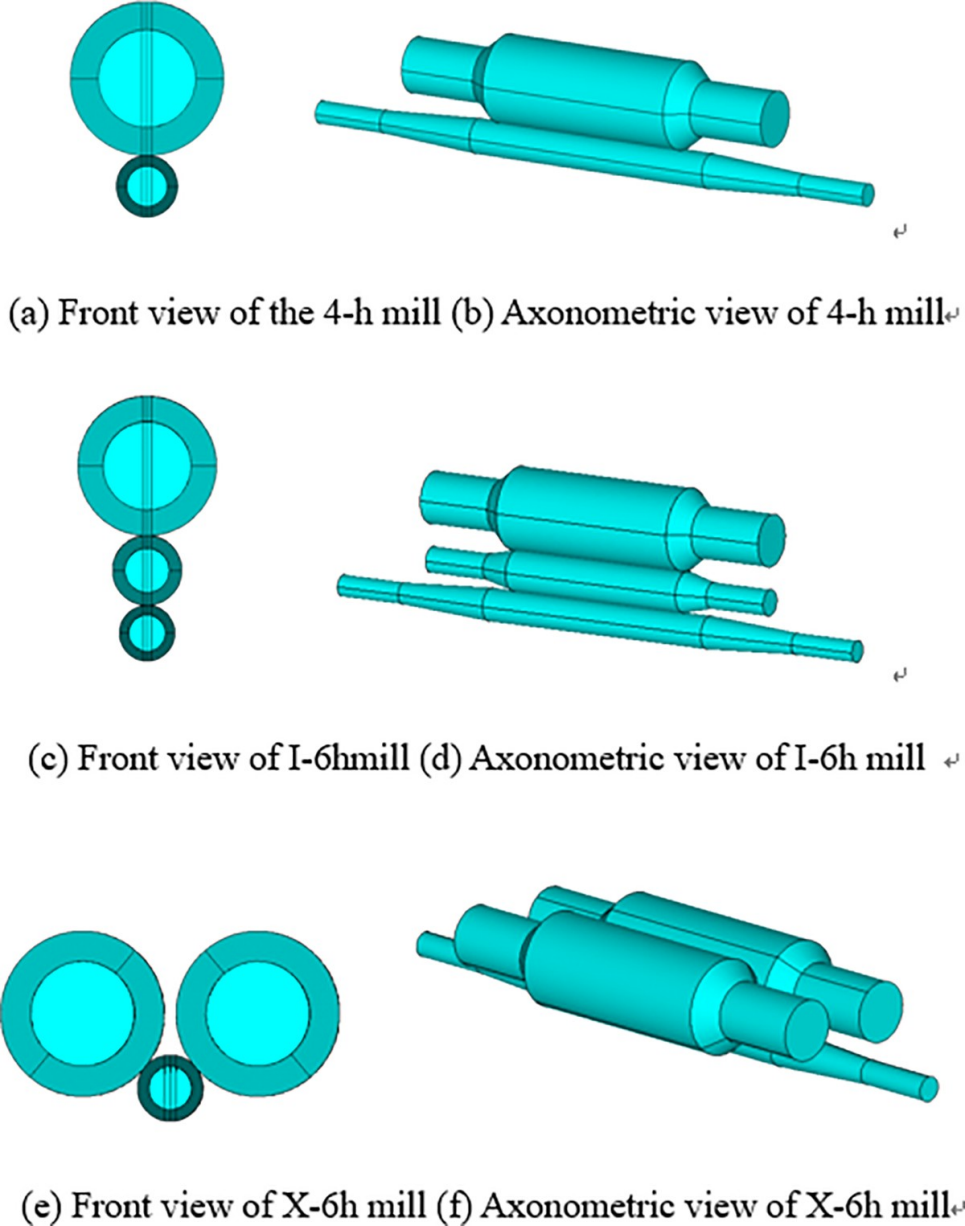
Simplified models of three kinds of roll layouts.

The 3D models of these three kinds of roll layouts are established, as shown in Fig 9. For the convenience of the present method, the simplifications are made by neglecting the following parameters of the rolls: 1) the position-limited edges (baffles); 2) the edge chamfers; 3) the center holes and bolt holes.

### 3.2 FEM modeling

**Fig 10:**
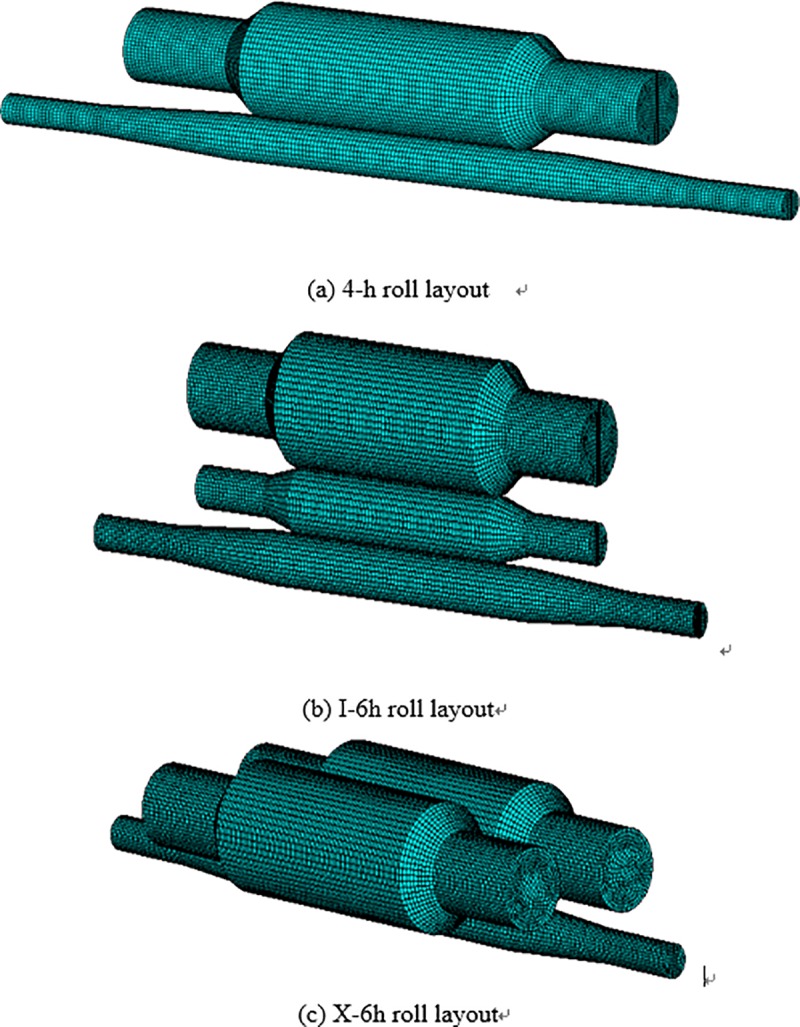
FE meshed models of the three different kinds of rolling mills.

An FE model is established using ANSYS software where the rolls are discretized by 8-node discrete elements SOLID45, and the material constants of the model are defined as: Young's modulus *E* = 2.06×10^5^ MPa, Poisson's ratio υ = 0.3. Overall meshing of the different roll layouts via sweeping method is conducted, and the grid size is set to 5 mm. The 4-h mill model consists of 76448 elements and 85455 nodes. The I-6h mill model consists of 104032 elements and 109974 nodes. The new X-6h mill model consists of 144600 elements and 151720 nodes. The meshed models are plotted in Fig 10.

**Fig 11:**
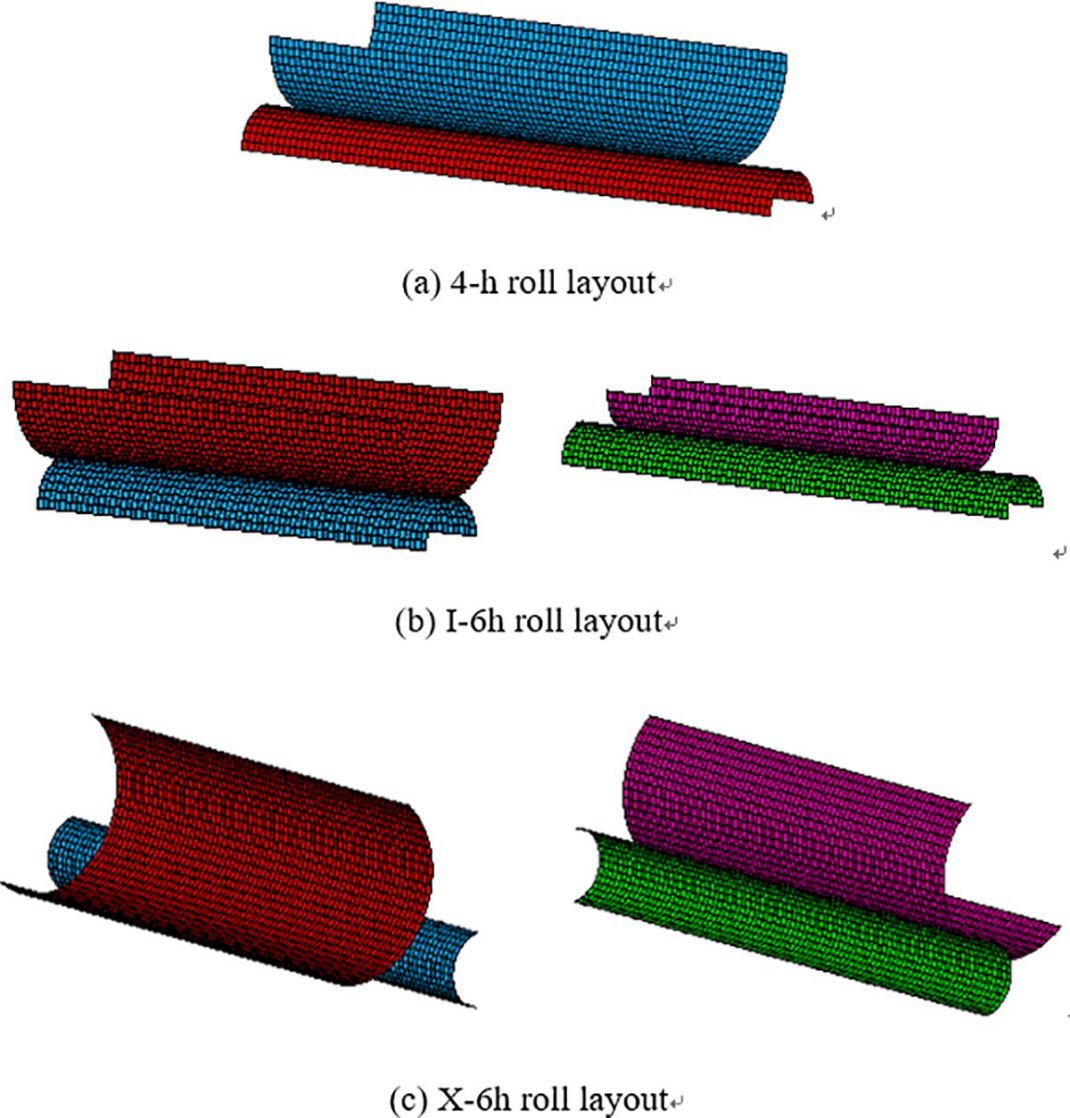
Contact pairs of the three kinds of roll layouts.

Subsequently, the contact pairs are set. When applying a face-to-face contact unit, one boundary is usually referred to as the "target" face, and the other boundary as the "contact" face. Typically, Targe 170 and Conta 174 are used to define 3D contact pairs as follows: one contact pair is set up for the 4-h roll system, which is the lower contact surface of UBUR and the upper target surface of UWR; two contact pairs are set for the I-6h roll system, which are the lower contact surface of UBUR and the upper target surface of UIMR, and the lower contact surface of UIMR and the upper target surface of UWR; two contact pairs are also set for the X-6h roll system, which are the right lower contact surface of ULBUR and the left upper target surface of UWR, and the left lower contact surface of URBUR and the right upper target surface of UWR. The established contact pairs for the three kinds of roll systems are shown in Fig 11.

**Fig 12:**
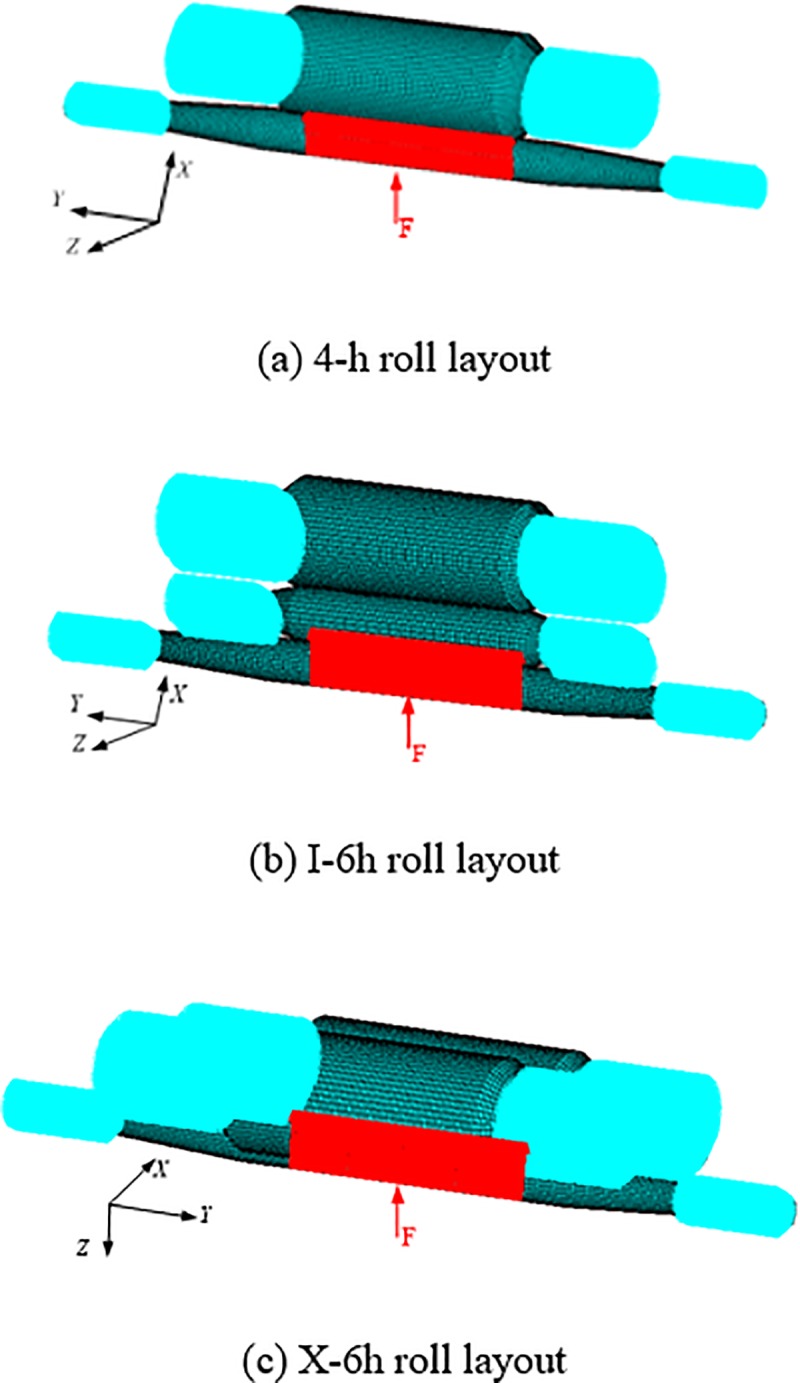
Constraints and loads attributed to the FEMs of the three rolling mills.

Considering the actual characteristics of the roll systems, reasonable simplification of constraints and loads can be carried out in finite element modeling. Because the BUR and the IMR are both tightly connected with the bearing in the shaft journal, they can be regarded as rigid constraints. That is to say, the contact surfaces between the bearing and shaft journal of BUR and IMR are regarded to be fully constrained in *x*, *y* and *z* directions. The contact surfaces between the bearing and shaft journal of WR are constrained in *y* and *z* directions. In Fig 12, the blue area is where constraints are applied. Here, the surface forces loaded on the model are determined by the stress exerted on the WR by the rolled material. Given a plate being 320 mm in width, all nodes (totally 325 nodes) at the bottom of the WR with a slice width of 320 mm are selected, a 200 N force is applied to each node, and the total load in the *x-*direction applied on one WR is 65 kN, as shown in Fig 12.

## 4. Numerical results and discussions

### 4.1 Comparative analysis of different roll layouts

In this section, the von Mises stress of the cross-section of the rolls, the contact stress between the rolls, and the displacement of each roll of the three kinds of roll layouts are calculated and analyzed. A vertical load 65 kN is applied on the WR, and the calculated results are listed in Table 2.

**Table 2:** Comparison of Stress and Displacement of Rolls with the Different Roll Layouts.

	4-h	I-6h	X-6h
**von Mises (MPa)**	28	34.9 (BUR) 38.6 (WR)	10.6
**Contact stress (MPa)**	74.5	91.6 (IMR and BUR) 78.3 (IMR and WR)	22.2
**Total displacement of BUR (μm)**	12.1	8.06	7.81
**Total displacement of IMR (μm)**	-	39.4	-
**Total displacement of WR (μm)**	46	83.4	12.3
**Total stiffness of roll system *K*** **(N/m)×10**^**9**^	1.4130	0.7794	5.2846

**Fig 13:**
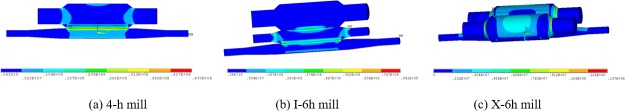
Von Mises stress field of the different roll layouts.

**Fig 14:**
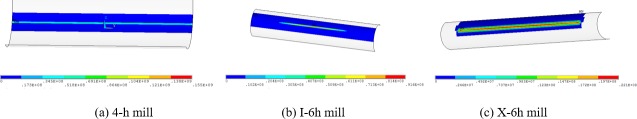
Contact stress field of the different roll layouts.

**Fig 15:**
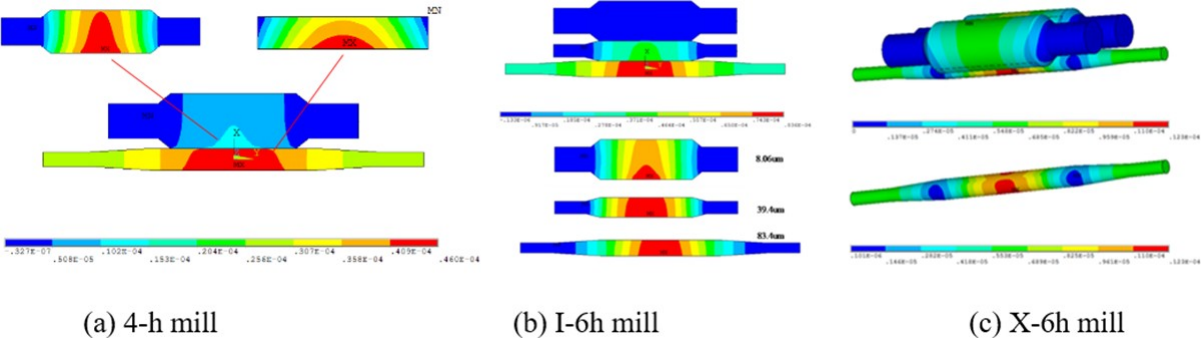
Displacement field of the different roll layouts.

It can be seen from Table 2 that under the same load conditions, the von Mises stress, contact stress of WR and BUR, and displacement of WR and BUR of these three kinds of roll layouts are greatly different. The von Mises equivalent stress, contact stress, WR displacement, and BUR displacement of the X-6h rolling mill are the smallest, followed by the 4-h mill lay, while the von Mises equivalent stress and contact stress of the I-6h mill are the largest. The contours of the Von Mises stress, contact stress, and displacement of the rolls of the different kinds of roll layouts are given in Figs 13–15.

### 4.2 Study on roll diameter ratios

Six sets of roll diameter ratios (0.3, 0.3158, 0.3333, 0.3529, 0.375, and 0.4) are selected for simulation. Keeping all other parameters unchanged, and the diameter ratios being changed, the effects of the different roll layouts on von Mises, contact stress and displacement of WR and BUR are studied. Table 3 shows stress and displacement of WR and BUR under different roll diameter ratios.

**Table 3:** Comparison of Stress and Displacement under Different Roll Diameter Ratios.

Item	Value					
*D*_1_/*D*_2_	0.3	0.3158	0.3333	0.3529	0.375	0.4
**von Mises** **(MPa)**	29.1	28.4	29.1	28.4	28.9	28
**Contact stress** **(MPa)**	76.9	76.5	76.2	75.7	75.3	74.5
**BUR *w*(*x*)** **(μm)**	7.25	8.12	8.41	9.31	10.1	12.1
**WR *w*(*x*)** **(μm)**	40.3	39.1	40.9	42.3	43.5	46
**Total stiffness of roll system *K*** **(N/m) ×10**^**9**^	1.6129	1.6624	1.5892	1.5366	1.4943	1.4130

From Table 3, it is observed that while the roll diameter ratios increase, the von Mises equivalent stress at the center of WR and BUR has little difference, the contact stress at the contact center of WR and BUR decreases, and the normal displacement of BUR and WR increases.

**Fig 16:**
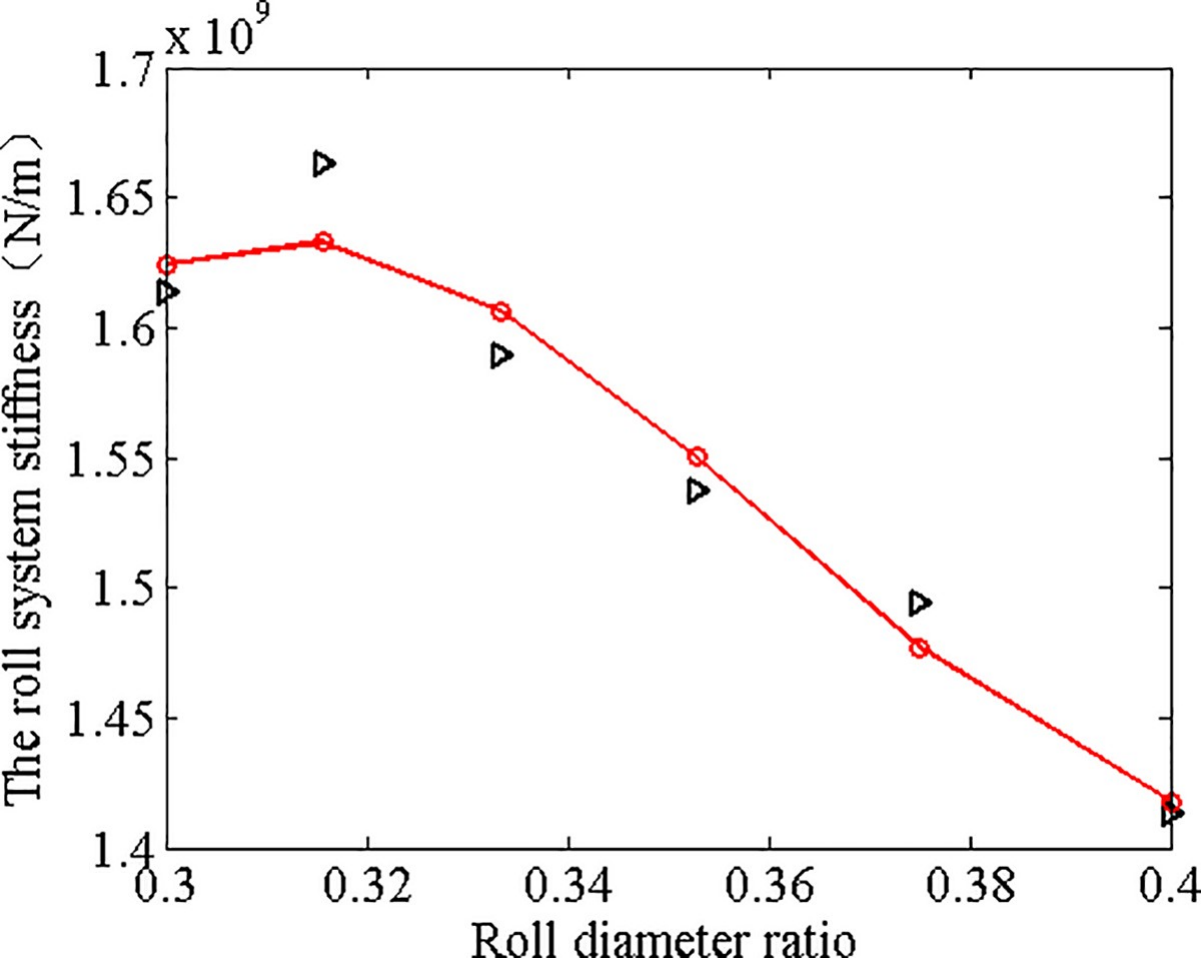
Relationship between roll system stiffness and roll diameter ratios.

The relationship between roll stiffness and roll diameter ratios can be drawn according to Table 3 (as shown in Fig 16), and the cubic fitting curve of roll system stiffness and roll diameter ratios can be obtained as follows:

$$K=10^{11}\times (4.5166x^{3}‐4.8960x^{2}+1.7354x‐0.1857)$$ (1)

## 5. Optimization design of X-6h roll system based on the genetic algorithm

The new X-6h mill has better stiffness characteristics than the other two types of mills. Furthermore, the structure of the X-6h rolling mill could be optimized based on an optimization theory, and on the basis of satisfying the requirements of stiffness and stress. The lightest roll system structure is recommended to design a good rolling mill. In this section, the agent model is used to establish the optimization model of the roll system. The stiffness of the roll system is taken as the optimization objective, the ratio of the roll diameter is selected as the design variable, and the optimization design is carried out based on GA.

### 5.1 Basic theory of the genetic algorithm

**Fig 17:**
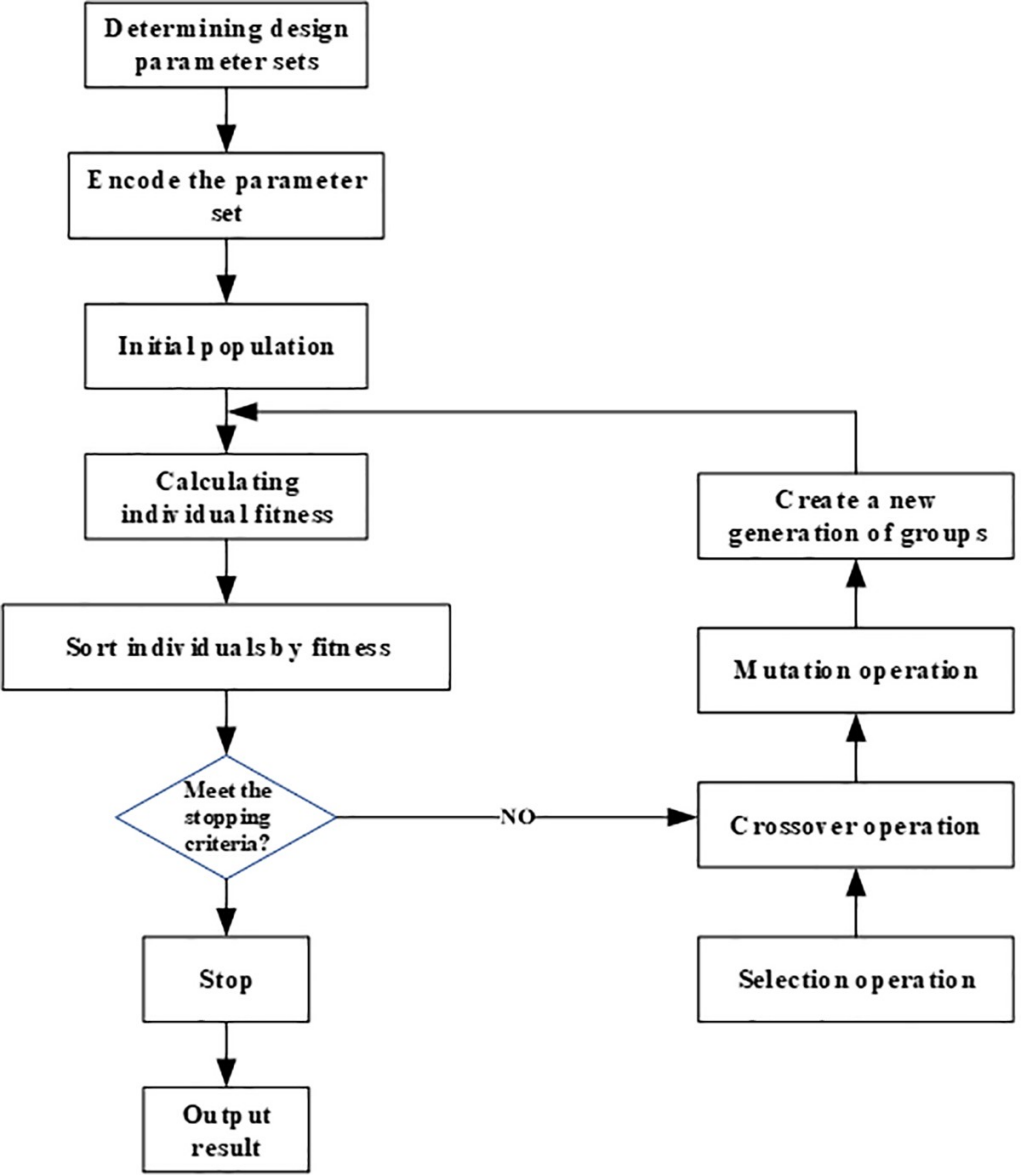
Flowchart of GA to optimize the stiffness of roll system.

The genetic algorithm uses the process of biological evolution to simulate the solution process of optimization problems, randomly initializes a population, and obtains the next generation population through operations such as copying and crossover. By comparing the values of the fitness function of each solution, eliminating the solution with low value, and increasing the solution with high value, the N-generation will evolve a solution with a high fitness function value. The main steps of the genetic algorithm include the design of fitness function, coding, selection, crossover, mutation and termination conditions. The flowchart for solving the optimization problem of the total stiffness of the roll system using GA is shown in Fig 17.

### 5.2 Optimization results

**Fig 18:**
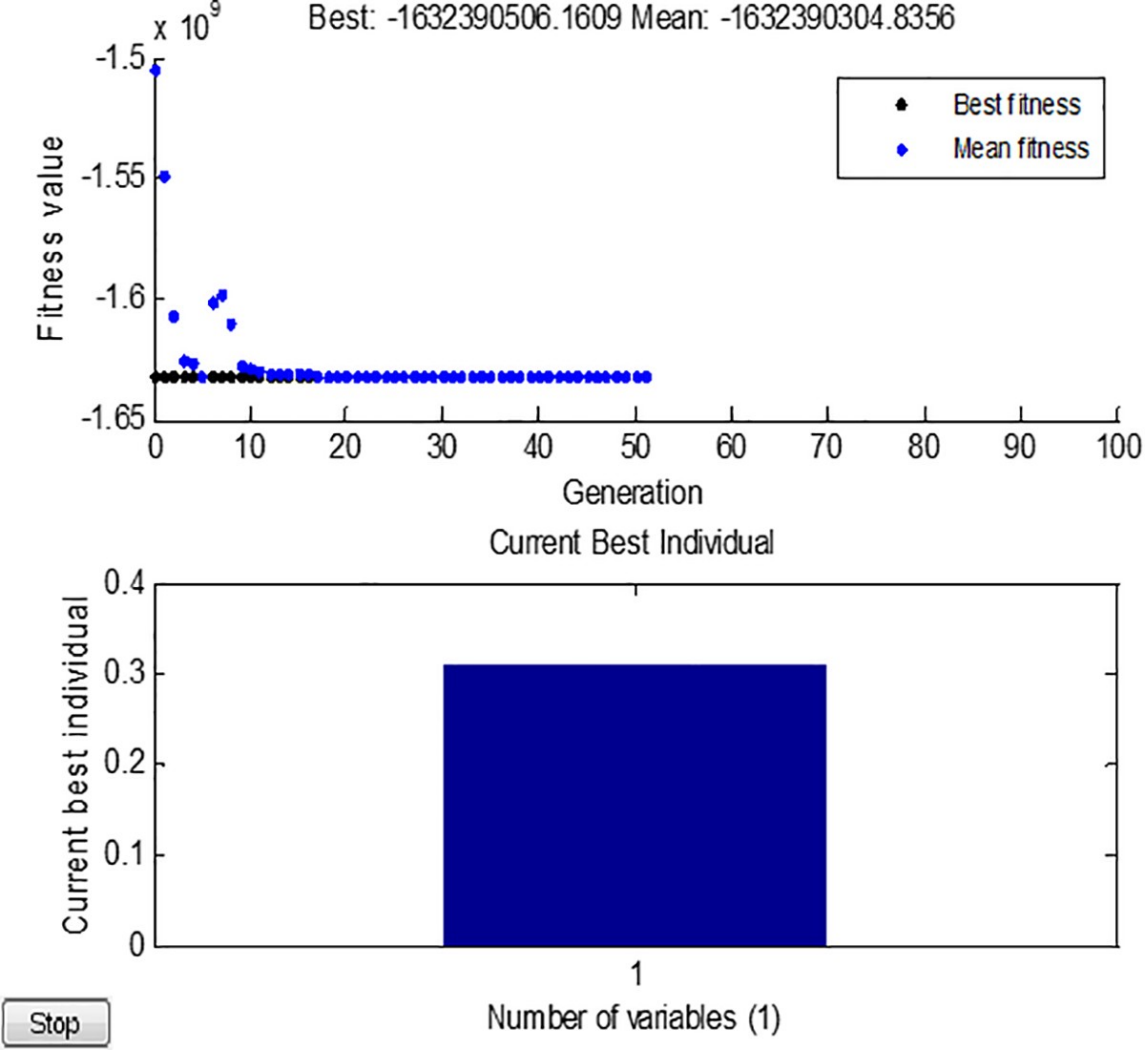
Iterative process of GA for optimizing roll diameter ratios.

The initial population is set to 200, the number of iterations to 100, the crossover operator to 0.8, and the mutation operator to 0.1, and the solution is optimized. The iterative process of optimizing roll diameter ratios by GA is shown in Fig 18.

**Fig 19:**
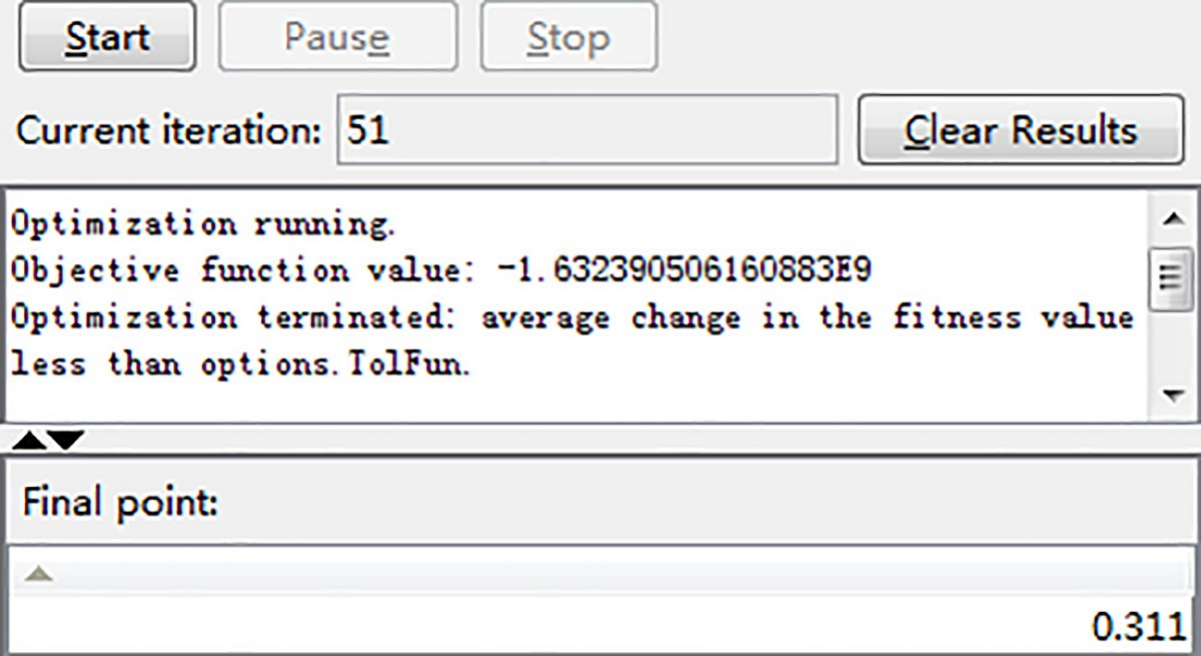
Iterative results of genetic algorithm for roll diameter ratios.

The specific iterative results are shown in Fig 19. The optimum roll ratio is 0.311, and the maximum stiffness is 1.6324×10^11^ N/m. The detailed roll size includes: the BUR diameter at 150 mm, and the WR diameter at 50 mm (150×0.311 = 46.65, take 50 mm).

## 6. Experimental verification

**Fig 20:**
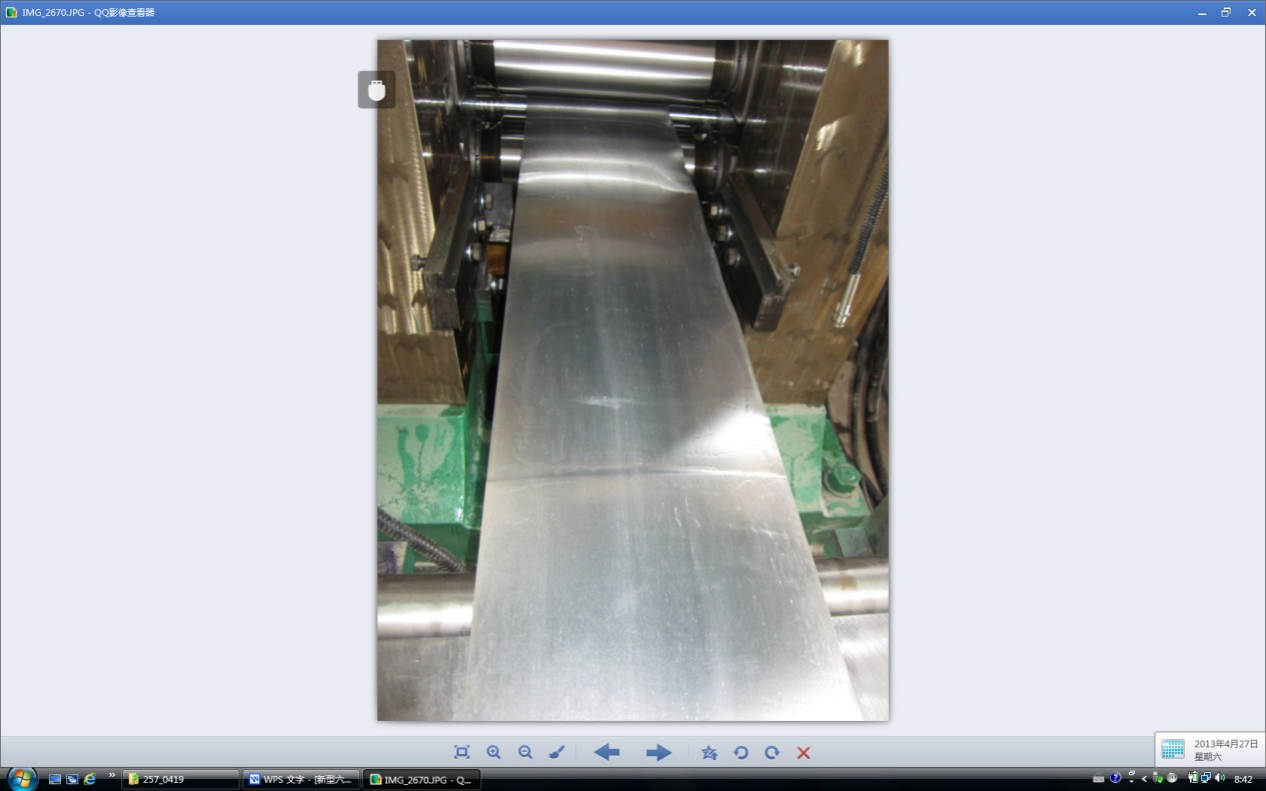
Magnesium alloy strip produced by X-6h rolling mill.

According to the results of the previous theoretical and computational analysis, a new X-6h rolling mill is designed, and as a matter of experience, the dimensions of BURs are determined to be 150 mm, and that of the WR is taken as 50 mm. The motors of the main driving and screw down systems are all selected according to the calculation results of rolling torque and pressure requirements. Additionally, the other standard components are chosen according to the mechanical design handbook. Finally, this set of equipment was manufactured by Wuxi Jingcheng Mechanical equipment industry, Jiangsu, P.R. China. Subsequently, the technical engineers helped with the installation. The rough materials were magnesium alloys manufactured by Yingkou Yinhe magnesium and aluminium Co.Ltd, and high precision magnesium alloy strips were successfully rolled by this machine, as shown in Fig 20.

**Fig 21:**
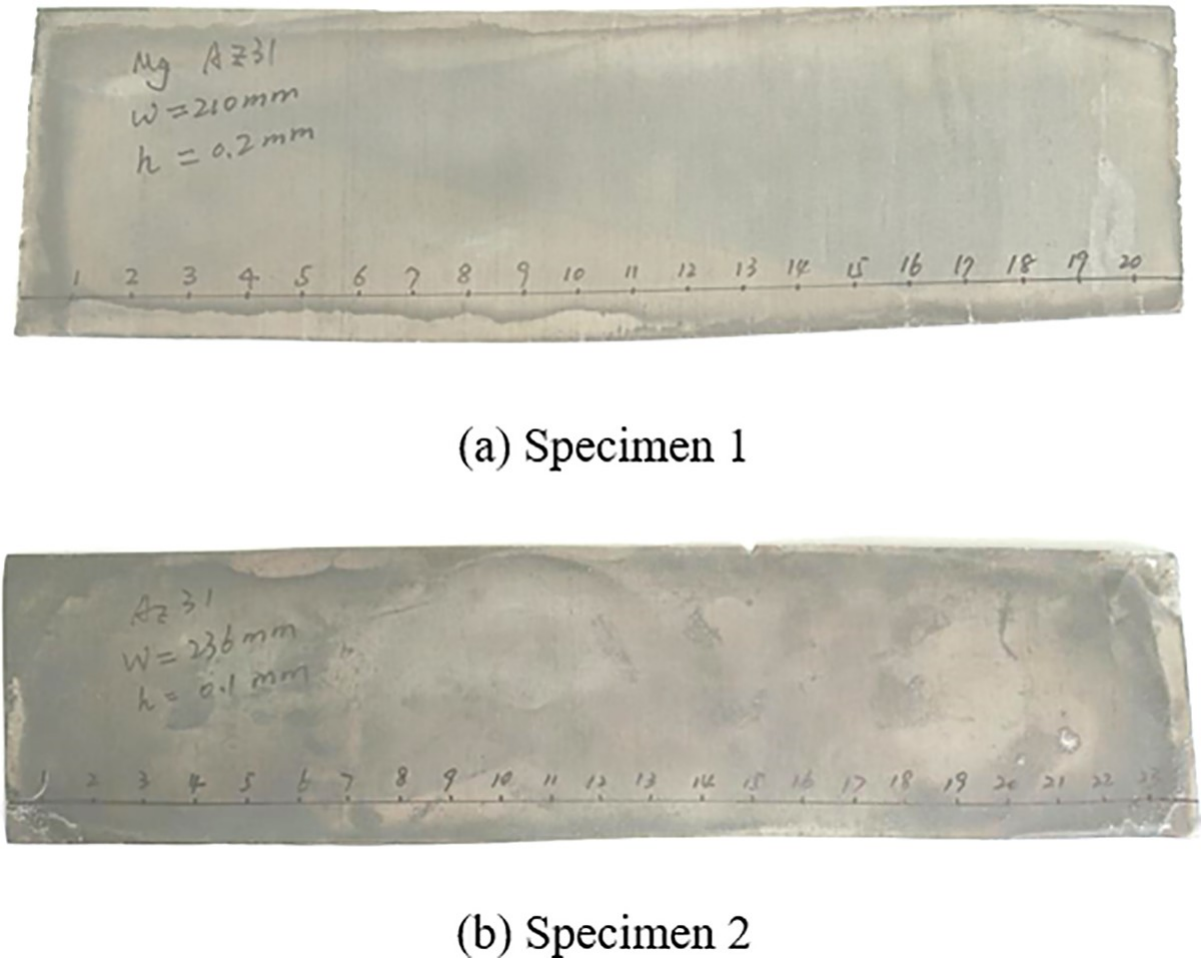
Magnesium alloy strips specimen.

Two pieces of AZ31 magnesium alloy strips were cut out, as shown in Fig 21, and the transverse thickness profile along the strip width direction of the specimen was measured.

**Fig 22:**
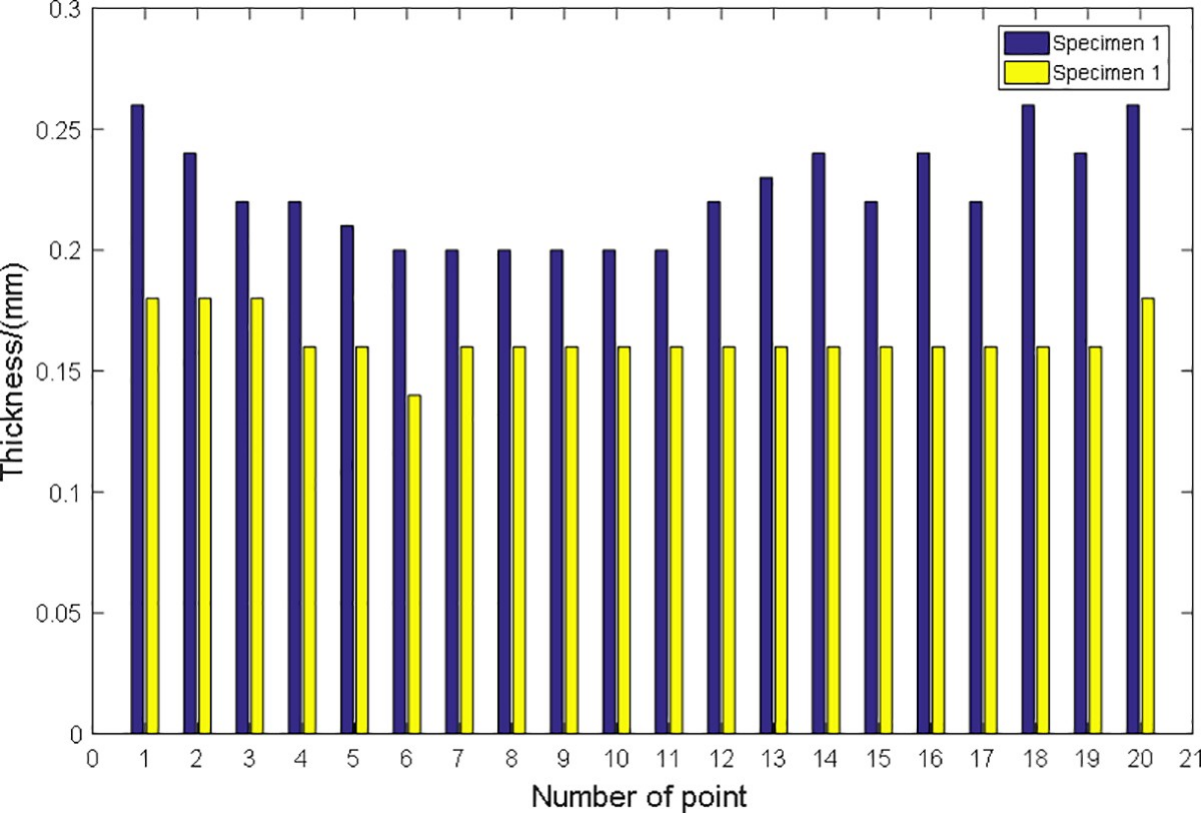
Histogram of transverse thickness profile along the strip width direction of specimens.

The transverse thickness profile along the strip width direction of the two strip specimens were measured separately, as shown in Table 4. The corresponding histogram is shown in Fig 22.

**Table 4:** Transverse Thickness Profile along the Strip Width Direction of Specimen.

Points	1	2	3	4	5	6	7	8	9	10	11	12	13	14	15	16	17	18	19	20
**Thickness of specimen 1 (mm)**	0.26	0.24	0.22	0.22	0.21	0.2	0.2	0.2	0.2	0.2	0.2	0.22	0.23	0.24	0.22	0.24	0.22	0.26	0.24	0.26
**Thickness of specimen 2 (mm)**	0.18	0.18	0.18	0.16	0.16	0.14	0.16	0.16	0.16	0.16	0.16	0.16	0.16	0.16	0.16	0.16	0.16	0.16	0.16	0.18

It can be seen from Fig 22, that the transverse thickness profile along the strip width of both specimen 1 and 2 is uniform, indicating that the rolled strips are of good quality with good flatness.

## 7. Conclusions

In this paper, a novel configuration of the X-6h rolling mill was proposed and its prototype machine manufactured. In addition, FEMs of different roll layouts, which were the 4-h mill, I-6h mill and X-6h mill, were respectively established, and by exerting contact pairs on adjacent rolls, the distribution of von Mises stress, contact stress, and elastic displacement of the WRs, IMRs, and BURs were analyzed. The influence of roll diameter ratios on the roll system stiffness was investigated, based on which an optimization design model was built, and then the optimum diameter ratios of BUR to WR of the X-6h rolling mill were achieved via the GA optimization method. Some detailed conclusions can be drawn as follows:

(1) As the demand for high-quality strips increases, contact stress distribution and roll deformation need to be further studied, and hence high-stiffness design for rolling mills is proposed in the rolling industry. By introducing contact pairs between rolls, Von Mises equivalent Stress, contact Stress and total Displacement of rolls were analyzed under the same load condition. The FEM results showed that the stress field distributions of the three roll layouts were basically the same. While the Von Mises equivalent stress, contact stress and total displacement of the X-6h WRs and BURs were lower than those of the 4-h and I-6h rolls, indicating that the proposed novel configuration of X-6h mill has better rigidity characteristic than that of the conventional 4-h and I-6h rolling mills.

(2) Six sets of roll diameter ratios of the WRs to BURs were selected to study the influence of diameter ratios on the roll system rigidity, and numerical simulation results showed that as the diameter ratios increased, the Von Mises stress at the contact center of WR and BUR had little difference, the contact stress between WR and BUR decreased, while the normal displacement increased. The relationship between roll system stiffness and roll diameter ratios was plotted and the cubic fitting equation obtained, based on which an agent model was built for the structural optimization of the X-6h rolling mill.

(3) Based on the cubic model of the roll system stiffness, the optimization analysis of the X-6h roll system stiffness was carried out by GA. The optimization results showed that when the ratio of roll diameter was 0.311, the stiffness was maximum, at 1.6324×1011 N/m. To verify the reliability of the proposed design and optimization method, a prototype rolling mill was manufactured according to optimized structural parameters, using which high-quality magnesium alloy and aluminum alloy strips with good flatness were produced. The results of this paper can provide reference for future research studies in related fields.

## References

[1] Wang QL, Li X, Hu YJ, Sun J, Zhang DH. Numerical analysis of intermediate roll shifting-induced rigidity characteristics of UCM cold rolling mill. Steel Research International. 2018;89(5). 10.1002/srin.201700454

[2] Aljabri A, Jiang ZY, Wei DB, Wang XD, Tibar H. Modeling of thin strip profile during cold rolling on roll crossing and shifting mill Proceedings of the 8th Pacific Rim International Congress on Advanced Materials and Processing. Springer, Cham 2013: 3001–3007.

[3] Wang DC, Liu HM, Liu J. Research and development trend of shape control for cold rolling strip. Chinese Journal of Mechanical Engineering. 2017;30(5):1248–1261. 10.1007/s10033-017-0163-8

[4] Wang D, Liu H. A model coupling method for shape prediction. Journal of Iron and Steel Research International. 2012;19(2):22–27. 10.1016/S1006-706X(12)60055-7

[5] Cao JG, Zhang J, Zhang SJ, 2010 Steel Rolling Equipment and Automation. Chemical Industry Press, Beijing.

[6] Cao JG, Chai Xt, Li YL, Kong N, Jia SH, Zeng W. Integrated design of roll contours for strip edge drop and crown control in tandem cold rolling mills. Journal of Materials Processing Technology. 2018;252:432–439. 10.1016/j.jmatprotec.2017.09.038

[7] Cavaliere MA, Goldschmit MB, Dvorkin EN. Finite element analysis of steel rolling processes. Computers and Structures. 2001;79(22–25): 2075–2089. 10.1016/S0045-7949(01)00055-4

[8] Hartley P, Sturgess C E N, Liu C, Rowe GW. Experimental and theoretical studies of workpiece deformation, stress, and strain during flat rolling. International Materials Reviews. 1989;34(1): 19–34. 10.1179/imr.1989.34.1.19

[9] Panton S M, Zhu SD, Duncan J L. Fundamental deformation types and sectional properties in roll forming. International Journal of Mechanical Sciences.1994;36(8):725–735. 10.1016/0020-7403(94)90088-4

[10] Komori Kazutake. Simulation of deformation and temperature in multi-pass three-roll rolling. Journal of Materials Processing Technology. 1999;92:450–7. 10.1016/S0924-0136(99)00175-2

[11] Sun CG, Hwang S M, Yun CS, Chung JS. Investigation of thermomechanical behavior of a work roll and of roll life in hot strip rolling. Metallurgical and Materials Transactions A.1998;29: 2407 10.1007/s11661-998-0117-y

[12] Dong Q, Cao JG. Contact Deformation analysis of elastic–plastic asperity on rough roll surface in a strip steel mill. Journal of Failure Analysis and Prevention. 2015;15(2):320–326. 10.1007/s11668-015-9936-5

[13] Shi X, Li SQ, Liu XH, Wang GD, Xu JY. FEM analysis for steel strip deformation in cold rolling process. Iron Steel. 2004;39:71–74. 10.1023/B:JOGO.0000006653.60256.f6

[14] Linghu KZ, Jiang ZY, Li F, Zhao JW, Yu M, Wang Y Q. FEM analysis of profile control capability during rolling in a 6-high CVC cold rolling mill. Advanced Materials Research. 2014;988:257–262. 10.4028/www.scientific.net/AMR.988.257

[15] Yasuda K, Narita K, Kobayashi K, Maeno I. Shape controllability in new 6-high mill (UC-4 mill) with small diameter work rolls. ISIJ International. 1991;31(6):594–598. 10.2355/isijinternational.31.594

[16] Aljabri A, Jiang ZY, Wei DB, Wang XD, Tibar H. Thin strip profile control capability of roll crossing and shifting in cold rolling mill. Materials Science Forum. Trans Tech Publications. 2014;773: 70–78.

[17] Mosayebi M, Zarrinkolah F, Farmanesh K. Calculation of stiffness parameters and vibration analysis of a cold rolling mill stand. The International Journal of Advanced Manufacturing Technology. 2017, 91(9–12): 4359–4369. 10.1007/s00170-017-0026-6

[18] Moon YH, Yi J J. Improvement of roll-gap set-up accuracy using a modified mill stiffness from gauge meter diagrams. Journal of Materials Processing Technology. 1997;70(1–3): 194–197. 10.1016/S0924-0136(97)02917-8

[19] Huang J L, Zang Y, Gao Z Y, Zeng L Q. Influence of asymmetric structure parameters on rolling mill stability. Journal of Vibroengineering.2017;19(7):4840–4853. 10.21595/jve.2017.18263

[20] Ma JH, Tao B, Yao XH. Multi-objective optimal design roller of Y-type mill using GA combined with FEM. Advanced Materials Research. 2014;898:225–228. 10.4028/www.scientific.net/AMR.898.225

